# Role of a new age-adjusted D-dimer cutoff value for preoperative deep venous thrombosis exclusion in elderly patients with hip fractures

**DOI:** 10.1186/s13018-021-02801-y

**Published:** 2021-10-30

**Authors:** Kexin Zhang, Yanbin Zhu, Yunxu Tian, Miao Tian, Xiuting Li, Yingze Zhang

**Affiliations:** 1 Department of Orthopaedic Surgery, The 3rd Hospital of Hebei Medical University, Shijiazhuang, 050051 Hebei People’s Republic of China; 2 Hebei Medical University, Shijiazhuang, 050017 Hebei People’s Republic of China; 3 Orthopaedic Institution of Hebei Province, Shijiazhuang, 050051 Hebei People’s Republic of China; 4 Key Laboratory of Biomechanics of Hebei Province, Shijiazhuang, 050051 Hebei People’s Republic of China

**Keywords:** Deep venous thrombosis, Age-adjusted D-dimer cutoff value, Hip fracture, Aged patients

## Abstract

**Objective:**

This study aimed to describe the characteristics of plasma D-dimer level with increasing age and establish a new age-adjusted D-dimer cutoff value for excluding preoperative lower limb deep vein thrombosis (DVT) in elderly patients with hip fractures.

**Methods:**

This was a retrospective study of elderly patients who presented with acute hip fracture in our institution between June 2016 and June 2019. All patients underwent D-dimer test and duplex ultrasound. Patients were divided into six 5-year-apart age groups. The optimal cutoff value for each group was calculated by using receiver operating characteristic (ROC) curves, whereby the new age-adjusted D-dimer cutoff value was determined. The sensitivity, specificity, positive predictive values (PPV) and negative predictive values (NPV) were calculated and compared when different D-dimer cutoff values were applied, i.e., conventional 0.5 mg/L, previously well-established age-adjusted cutoff value (age × 0.01 mg/L) and the new age-adjusted D-dimer cutoff value herein.

**Results:**

There were 2759 patients included, 887 males and 1872 females, with an average age of 78 years. In total, 280 patients were diagnosed with preoperative DVT. The optimal cutoff values for the six age groups were 0.715 mg/L, 1.17 mg/L, 1.62 mg/L, 1.665 mg/L, 1.69 mg/L and 1.985 mg/L, respectively, and the calculated age-adjusted coefficient was 0.02 mg/L. With this new coefficient applied, the specificity was 61%, clearly higher than those for conventional threshold (0.5 mg/L, 37%) or previously established age-adjusted D-dimer threshold (age × 0.01 mg/L, 22%). In contrast, the sensitivity was lower than that (59% vs 85% or 77%) when D-dimer threshold of 0.5 mg/L or age-adjusted cutoff value (age × 0.01 mg/L) was used. The other indexes as PPV (15%, 11% and 12%) and NPV (93%, 93% and 94%) were comparable when three different D-dimer thresholds were applied.

**Conclusions:**

We developed a new age-adjusted D-dimer cutoff value (age × 0.02 mg/L) for a specified high-risk population of patients aged 65 years or older with hip fractures, and demonstrated the improved utility of the D-dimer test for exclusion of DVT. This formula can be considered for use in elderly hip fracture patients who meet the applicable standards as preoperative DVT screening, after its validity is confirmed by more well-evidenced studies.

## Introduction

Deep vein thrombosis (DVT) is a common potentially fatal disorder, with a variable prevalence of 4 to 52% [1–5]. Prompt diagnosis and targeted treatment as the most major methods to reduce the risk of proximal DVT migration or pulmonary embolism (PE), and even death, are still in progress [6]. The D-dimer test, as an important link of the DVT diagnostic algorithm, is generally used as an initial screening tool in large population because of its simple operation and high sensitivity. However, its low specificity increases additional medical burden [7]. Therefore, how to improve these two dilemmas has become the main problem faced by researchers. In order to improve the specificity and reduce unnecessary expenditure on medical resources, the age-adjusted D-dimer cutoff values and combination diagnosis trials have been consistently the research focuses in various medical fields or in different settings during the past decade [8, 9]. D-dimer as a diagnostic biological marker of DVT, is affected not only by age but also by trauma from fracture or surgery [10]. Fractures associated with hypercoagulability of blood, trauma, immobility, hospitalization, and inflammatory immune response of the body put patients at a high risk of DVT [11, 12]. Hip fractures presented with a substantially higher incidence rates of 17–58% for preoperative DVT than those of distal limb fractures, such as tibiofibular or plateau fractures (12%), and ankle fractures (6%), calcaneal fractures (12%), and further had the significantly increased risk of proximal thrombosis, PE, and mortality [10, 13–16].

A considerable number of studies have re-adjusted D-dimer level associated with age in patients with venous thromboembolism (VTE) [17–19]. Douma et al. [20] proposed and established the typical age-adjusted D-dimer threshold (age × 0.01 mg/L) to improve specificity. In the past decade, the use of age-adjusted D-dimer threshold (age × 0.01 mg/L) has demonstrated the improved specificity in diagnosis of DVTs, aiding in exclusion of those with no thromboembolism in most cases [21–24]. For example, Dutton et al. [25] used the age-adjusted D-dimer threshold (age × 0.01 mg/L) for the diagnosis of PE, with specificity increasing from 7 to 32%.

In order to reduce the risk of complication, hip fractures among older people should be operated on within 24–48 h of hospital admission in many medical centers [26]. Similarly, in most cases, hip fracture as a major trauma is a high-risk factor for DVT [11]. Routine deep vein examinations of the lower extremities are often required to exclude thrombosis, thereby reducing the risk of thromboembolism, especially PE, and even death. From the time point of view, the two are most likely to be contradictory. It is difficult to complete the routine check of DVT and early surgery in such a short time as 24–48 h. Second, as a very sensitive biochemical indicator for detecting DVT, the sensitivity of D-dimer is up to 95%, but its specificity is very low, especially for elderly patients, which are precisely the population with a high incidence of hip fractures [22], additionally, hip fracture itself is a high-risk related factor for D-dimer, the two factors together make the specificity of D-dimer extremely low, which greatly reduces its clinical value. Some researchers even do not recommend D-dimer testing was used in the elderly [27]. Therefore, it is very necessary to improve the diagnostic performance of D-dimer test, especially the specificity.

Therefore, the study aims to investigate the age-dependent characteristics of D-dimer in aged (≥ 65 years) patients who had hip fractures, and second to establish a new age-adjusted D-dimer cutoff value and evaluate its ability to safely exclude elderly hip fracture patients without DVT.

## Methods

### Study population

**Fig. 1:**
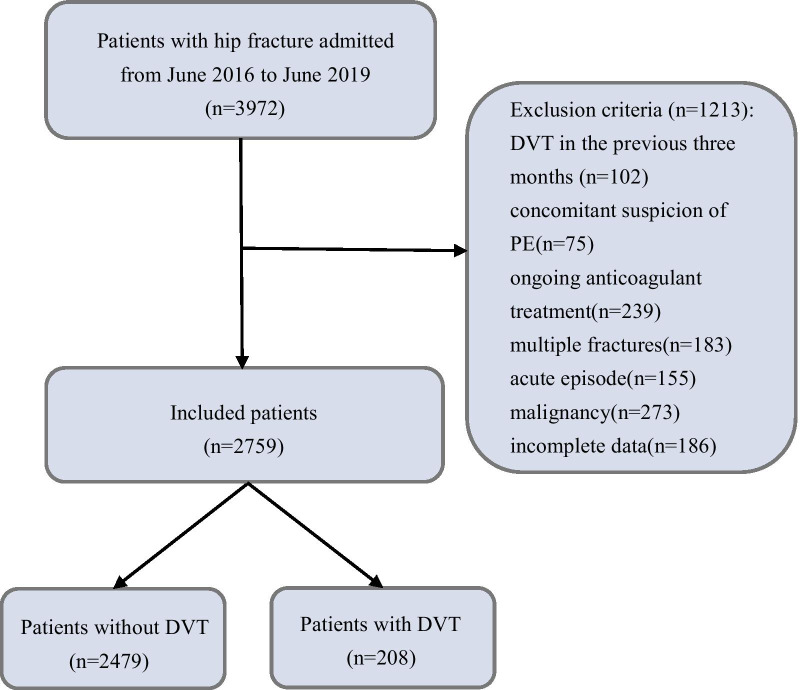
Flow diagram of the study. Of 3972 aged patients with hip fracture admitted to hospital. 1213 patients who did not meet the inclusion criteria were excluded. Of the 2759 left. Of those patients, 2479 did not had DVT and 280 did

In this study, data on 2759 patients with the diagnosis of acute hip fractures who were surgically treated at the 3rd Hospital of Hebei Medical University between June 2016 and June 2019, were retrospectively collected. All patients who adhered to the following criteria were included: age ≥ 65 years and experiencing both D-dimer test and DUS preoperatively. D-dimer level is susceptible to various factors from trauma, age, malignancy, acute hemodynamically instable events, and the previous history of VTE or the current anti-coagulation medications [7, 24, 28]. For ruling out their strong impact of these driving factors, we pre-defined the more stringent criteria. Patients were excluded if they had suffered a VTE within the three months before the index hip fracture, high-energy accident, long-term injured-limb immobility or the significant delay to admission, concomitant suspicion of PE, ongoing anticoagulant treatment, multiple fractures, acute episode (acute infection, acute heart failure, etc.), malignancy, incomplete data. The demographic data (age and sex) were collected from the clinical medical records. The D-dimer test results and DUS results were extracted from the laboratory department and the imaging department, respectively. If there were multiple preoperative examinations for one patient, we only selected the initial result to analyze. The flow diagram for the patient selection is shown in Fig. 1.

### Intervention

During the inpatient admission, all patients received basic pharmacological thromboprophylaxis (low molecular weight heparin (LMWH), 2500–4100 IU once daily, subcutaneous injection). For patients diagnosed with DVT, the therapeutic doses of anticoagulant drugs (low molecular heparin (LMWH), 2500–4100 IU twice daily, subcutaneous injection) were taken.

### Study design

Following Douma’s strategy [20] to establish an age-adjusted D-dimer cutoff value, we attempted to explore a new age-adjusted D-dimer cutoff value to increase the proportion of elderly hip fracture patients in whom a preoperative DVT could be safely excluded. We divided all patients into six 5-year-apart age groups (65–69, 70–74, 75–79, 80–84, 85–89 and above or equal to 90 years). The receiver operating characteristic (ROC) curves were subsequently constructed to determine the optimal cutoff value (defined as the value at which the Youden index is at its maximum) for each group. The simple linear regression model was used to calculate regression coefficients based on the six optimal cutoff values in age groups. The calculated coefficient corresponded to an increase in the D-dimer cutoff value per five years. To calculate the annual increase in the D-dimer cutoff value, the regression coefficient was divided by 5 (the number of years per 5 years). This coefficient was used as a multiplier of the patient's age when determining the new age-adjusted D-dimer cutoff value.

### Diagnostic procedure

D-dimer was detected following admission in all patients in the morning. The blood samples were collected on an empty stomach and in a quiet state and sent to the central laboratory for testing on the Wondfo FS-301 Auto-Immunofluorescence Quantitative Analyzer (Xiamen, China) within 60 min. The D-dimer results were categorized into negative group or positive group based on the manufacturers' cutoff value (0.5 mg/L).

Before surgery, a complete DUS examination was performed with Philips Affiniti 50 ultrasonographic machine (Royal Philips Electronics, Amsterdam, Netherlands) in all patients. The detection areas consisted of femoral vein, popliteal vein, calf vein and peroneal and tibial veins. The DUS examination was performed by technicians with professional qualification certificates and without knowledge of the patients' D-dimer test results. The DVT diagnosis result was based on the Robinov group's criteria [29], as follows: there were incompletely compressible vein, intraluminal thrombus or filling defect and poor in phasic vibration with respiratory movements of calf compression. Thrombosis can be diagnosed if any of the above two or more ultrasound criteria were confirmed. All lower limb veins were scanned as many as possible by registered technicians.

### Outcomes

The primary outcome was the establishment of the new age-adjusted cutoff value and the diagnostic parameters of three different thresholds including specificity, sensitivity, negative predictive value (NPV) and positive predictive value (PPV), negatives number, false negatives number, number needed to test (NNT). The secondary outcome was the average value of D-dimer and the incidence of DVT for each age group to verify the increasing trend of D-dimer with age.

### Statistical analysis

Continuous variables were described as mean ± standard deviation; categorical variables were expressed by the number and percentages (%). Kruskal–Wallis test was used for the comparison of multiple groups of non-normally distributed data. Chi-square test was used to compare the specificity and sensitivity among three different groups. *P* < 0.05 was considered significant. The ROC analysis was subsequently constructed to determine the optimal cutoff value. The simple linear regression analysis was used to calculate regression coefficients, SPSS 26.0 was used for analysis and Graph Pad Prism 9 software was used to draw figures.

### Ethics approval

The study was carried out adhering to the Helsinki Declaration consensus and was approved by the institutional review board. Informed consent was waived for this retrospective review as no identifying information was recorded.

## Result

### Characteristics of study subjects and D-dimer blood level

**Fig. 2:**
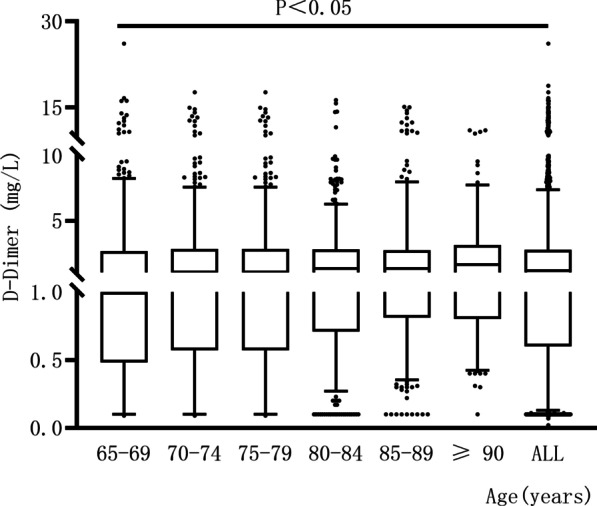
The D-dimer level of all patients and six 5-year age groups. The level of D-dimer in each group was shown as box plots with a percentile of 5–95%. Comparison of 7 groups: *p* < 0.05

Our study included 2759 elderly patients who had hip fractures. Of these, 887 were male and 1872 were female, with a mean age of 78 years. In this series, 280 (10%) patients were diagnosed with DVT using DUS. The level of D-dimer increased consecutively with age from 2 mg/L in the age group 65–69 to 2.36 mg/L in the age group ≥ 90. The difference was statistically significant (*P* < 0.05). The D-dimer level was 2.36 mg/L in DVT group higher than 2.11 mg/L in non-DVT group. The variation of D-dimer levels with age, specific gender distribution, mean age, D-dimer level mean, and prevalence of DVT in each age group are presented in Table 1 and Fig. 2.

**Table 1:** Baseline characteristics of 2759 patients

	65–69	70–74	75–79	80–84	85–89	≥ 90	All ages
Number n (%)	495 (18)	518 (19)	555 (20)	611 (22)	407 (15)	173 (6)	2759 (100)
Female sex n (%)	325 (66)	356 (69)	371 (67)	411 (67)	287 (71)	122 (71)	1872 (68)
Age (mean ± SD)	67.12 ± 1.39	72.00 ± 1.41	77.14 ± 1.40	81.88 ± 1.40	86.72 ± 1.39	92.19 ± 2.55	77.78 ± 7.59
D-dimer mean in patients with DVT (mg/L)	2.1	2.2	2.3	2.4	2.5	3.4	2.4
D-dimer mean in patients without DVT (mg/L)	1.98	2.09	2.1	2.11	2.26	2.27	2.11
D-dimer mean in all patients (mg/L)	2.00	2.10	2.12	2.13	2.28	2.36	2.14
DVT N (%)	55 (11)	45 (9)	70 (13)	57 (9)	38 (9)	15 (9)	280 (10)

*SD* standard deviation, *DVT* Deep venous thromboembolism

### Establishment of a new age-adjusted D-dimer cutoff value

**Fig. 3:**
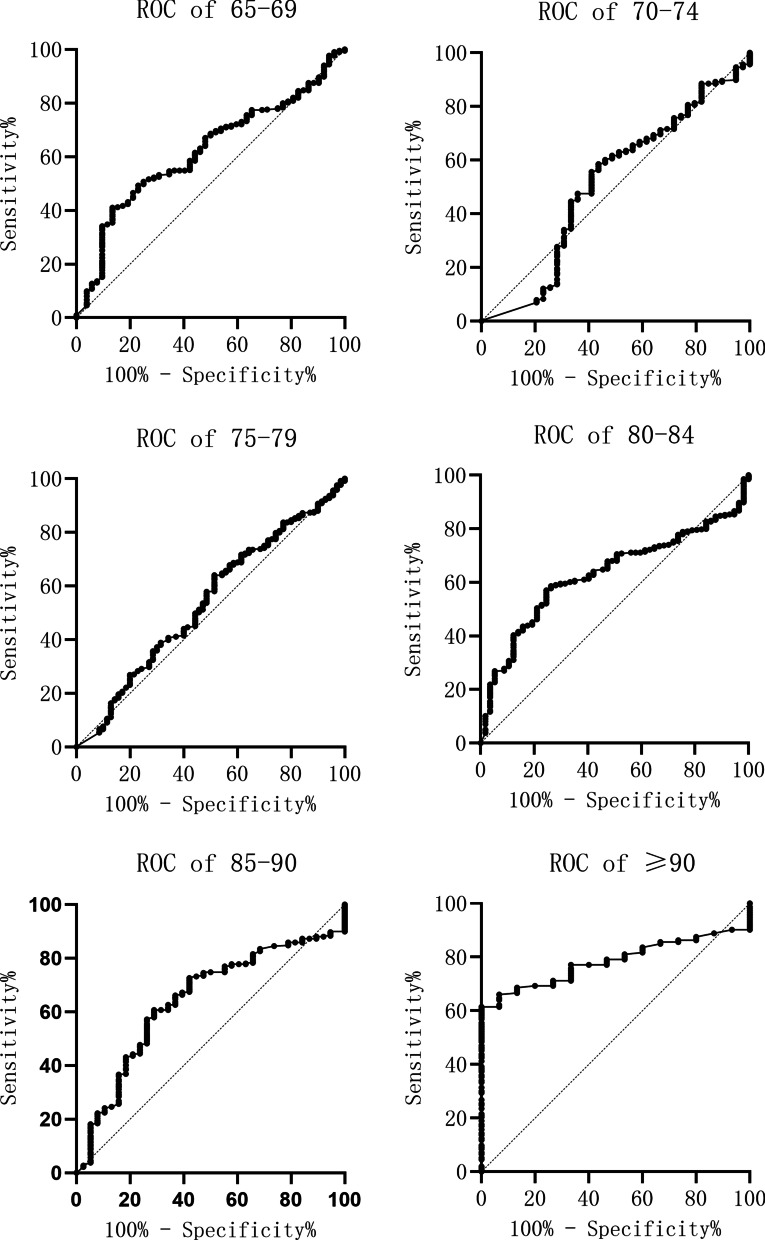
Receiver operating characteristic (ROC) curves of the D-dimer test for each 5-year-apart age group. The optimal cutoff value for each group was determined when the Youden index was at its maximum

**Fig. 4:**
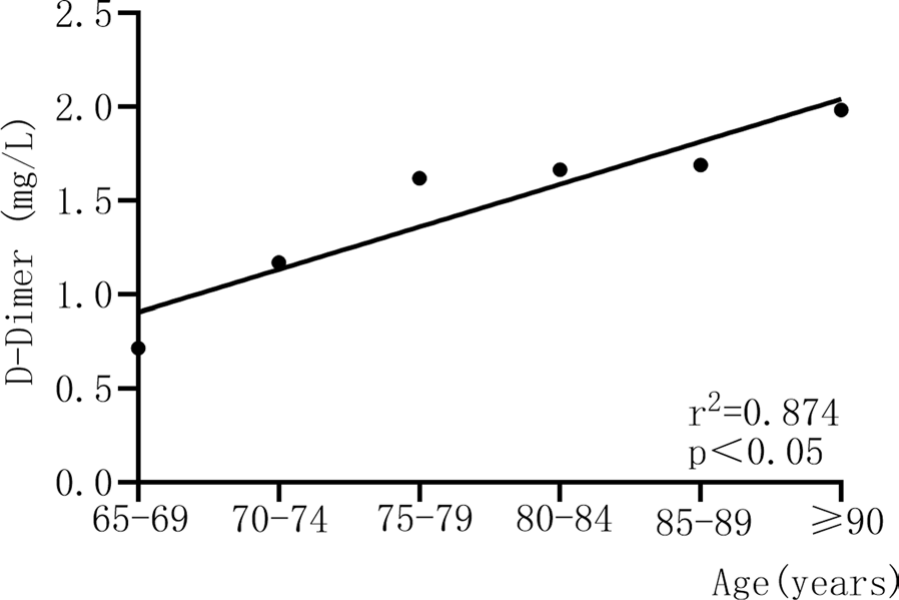
Linear regression analysis of optimal cutoff value and 5-year age groups. Through linear regression analysis, the regression coefficient (r, corresponding to the slope of the regression line) was determined (here: 0.23; 95% confidence interval 0.11–0.35). This coefficient represented an increase in the level of D-dimer per 5 years. By dividing the regression coefficient by 5, the annual increase in the level of D-dimer was obtained. In determining the new age-adjusted D-dimer threshold, the calculated coefficient was used as a multiplier for the patient's age

According to the ROC curves, the optimal thresholds of the six 5-year-apart age groups were 0.715 mg/L, 1.17 mg/L, 1.62 mg/L, 1. 665 mg/L, 1.69 mg/L and 1.985 mg/L in turn, as they are shown in Fig. 3. The optimal D-dimer cutoff values of each age group were plotted by simple linear regression analysis. Thereby, the regression coefficient (*r*) corresponding to the slope of the regression line was calculated as 0.23 mg/L (95% confidence interval 0.11 to 0.35), as shown in Fig. 4. To ensure maximum sensitivity, we chose the lower limit 0.11 mg/L (5% CL value) instead of 0.23 mg/L as regression coefficient. This coefficient represented the increase in the D-dimer cutoff value per five years. The annual increase in the D-dimer cutoff value was obtained by dividing the regression coefficient (0.11 mg/L) by 5. The coefficient of 0.02 mg/L was used as a multiplier of the patient's age when determining the new age-adjusted D-dimer cutoff value. All procedures are summarized in Figs. 3 and 4.

### Comparison of different D-dimer cutoff value

Among 2759 patients, 594 (22%) had a D-dimer level of < 0.5 mg/L (traditional threshold), 979 (35%) and 1293 (59%) patients had a lower dichotomized D-dimer level, based on the previously established age-adjusted cutoff values of age × 0.01 mg/L and the new age-adjusted cutoff value of age × 0.02 mg/L. At the threshold of 0.5 mg/L, the sensitivity was 85% (95% confidence interval [CI], 80.1–88.9) with a specificity of 22% (95% CI, 20.7–24.0), PPV of 11% (95% CI, 9.7–12.4) and NPV of 93% (95% CI, 90.5- 94.8) in all ages. At the age-adjusted cutoff value (age × 0.01 mg/L), sensitivity was 77% (95% confidence interval [CI], 71.7–81.8) with a specificity of 37% (95% CI, 35.0–38.8), PPV of 12% (95% CI, 10.7–13.8) and NPV of 94% (95% CI, 91.7- 94.9) in all ages. At the new age-adjusted cutoff value (age × 0.02 mg/L), sensitivity was 59% (95% confidence interval [CI], 52.9–64.7) with a specificity of 61% (95% CI, 59.3–63.2), PPV of 15% (95% CI, 12.7–16.9) and NPV of 93% (95% CI, 91.6–94.1) in all ages. The NNT to find one normal D-dimer test result was 4.2 at the threshold of 0.5 mg/L and 2.5 at the age-adjusted cutoff value (age × 0.01 mg/L) and 1.5 at the new age-adjusted cutoff value (age × 0.02 mg/L). In contrast, the sensitivity was lower than that (59% vs 85% or 77%) when D-dimer threshold of 0.5 mg/L or age-adjusted cutoff value (age × 0.01 mg/L) was used. All results are reported in Table 2.

**Table 2:** Evaluation parameters in three different strategies

	Negative number *n* (%)	Number needed to test	False negatives number (%)	Positive predictive value (%, 95% CI)	Negative predictive value (%, 95% CI)	Sensitivity (%, 95% CI)	Specificity (%, 95% CI)
0.5	594 (22)	4.2	42 (15)	11 (9.7, 12.4)	93 (90.5, 94.8)	85 (80.1, 88.9)	22 (20.7, 24.0)
0.01*age	979 (35)	2.5	64 (23)	12 (10.7, 13.8)	94 (91.7, 94.9)	77 (71.7, 81.8)	37 (35.0, 38.8)
0.02*age	1293 (59)	1.5	115 (41)	15 (12.7, 16.9)	93 (91.6, 94.1)	59 (52.9, 64.7)	61 (59.3, 63.2)
*P*	–	–	–	–	–	< 0.05	< 0.05
0.5	148 (30)	3	8 (15)	14 (10.2, 17.7)	95 (89.3, 97.5)	86 (72.8, 93.1)	32 (27.5, 36.4)
0.01*age	183 (37)	2.4	10 (18)	14 (10.8, 18.9)	95 (89.9, 97.2)	82 (68.6, 90.5)	39 (34.8, 44.1)
0.02*age	291 (59)	1.5	26 (42)	14 (9.9, 19.9)	91 (87.0, 94.0)	53 (38.9, 66.1)	60 (55.5, 64.8)
*P*	–	–	–	–	–	< 0.05	< 0.05
0.5	166 (32)	2.8	17 (38)	8 (5.4, 11.4)	90 (83.9, 93.7)	62 (46.5, 75.8)	32 (27.4, 35.9)
0.01*age	221 (43)	2.1	19 (42)	9 (5.9, 12.7)	91 (86.7, 94.6)	58 (42.2, 72.0)	43 (38.2, 47.3)
0.02*age	335 (65)	1.4	26 (58)	10 (6.5, 16.0)	92 (88.7, 94.8)	42 (28.0, 57.8)	65 (60.8, 69.6)
*P*	–	–	–	–	–	< 0.05	< 0.05
0.5	121 (22)	4	13 (19)	13 (10.2, 16.8)	89 (82.0, 93.9)	81 (70.0, 89.4)	22 (18.7, 26.3)
0.01*age	208 (37)	2.3	22 (31)	13 (10.5, 18.0)	89 (84.2, 93.1)	69 (56.2, 78.9)	38 (34.0, 42.9)
0.02*age	340 (61)	1.4	36 (51)	16 (11.3, 21.5)	89 (85.5, 92.4)	49 (36.6, 60.7)	63 (58.2, 67.0)
*P*	–	–	–	–	–	< 0.05	< 0.05
0.5	97 (16)	5.7	2 (4)	10 (7.4, 12.5)	94 (82.8, 98.5)	95 (84.5, 98.)	9 (6.5, 11.4)
0.01*age	189 (31)	2.9	7 (12)	11 (8.1, 14.8)	93 (89.2, 96.0)	72 (58.3, 82.6)	40 (36.3, 44.7)
0.02*age	332 (54)	1.7	14 (24.56)	15 (11.5, 20.3)	96 (92.9, 97.6)	75 (62.0, 85.5)	57 (53.2, 61.5)
*P*	–	–	–	–	–	< 0.05	< 0.05
0.5	44 (11)	8.4	2 (5.26)	10 (7.1, 13.6)	96 (83.3, 99.2)	95 (80.9, 99.1)	11 (8.4, 15.2)
0.01*age	120 (29)	3.1	6 (15.79)	11 (7.9, 15.5)	95 (89.0, 98.0)	84 (68.1, 93.4)	31 (26.3, 35.9)
0.02*age	240 (59)	1.5	13 (34.21)	15 (10.1, 21.5)	95 (90.7, 97.0)	66 (48.6, 79.9)	62 (56.3, 66.5)
*P*	–	–	–	–	–	< 0.05	< 0.05
0.5	18 (10)	8.8	0 (0.00)	10 (5.7, 15.7)	100 (78.1, 100.0)	100 (74.7, 100.0)	11 (7.1, 17.7)
0.01*age	58 (34)	2.7	(0.00)	13 (7.7, 20.9)	100 (92.3, 100.0)	100 (74.7, 100.0)	37 (29.3, 44.8)
0.02*age	96 (55)	1.6	0 (0.00)	20 (11.7, 30.4)	100 (95.2, 100.0)	100 (74.7, 100.0)	61 (52.7, 68.3)
*P*	–	–	–	–	–	> 0.05	< 0.05

Number needed to test to find one normal D-dimer test result

## Discussion

Our findings are consistent with published research showing that D-dimer levels increase with age. The new age-adjusted D-dimer cutoff value (age × 0.02 mg/L) significantly improves the specificity of the D-dimer assay, from 22 to 61%, and reduces the NNT to find one normal D-dimer test result to 1.5. When the new threshold is used, the proportion of patients in whom DVT could be safely ruled out among the six 5-year-apart age groups is between 52 and 60%, increasing by 66% when compared to the typical cutoff value of age × 0.01 mg/L.

In the setting of both advanced age and a major trauma, the risk of DVT was higher than general population or other conditions, and investigation of targeted prompt examination method remains a key topic [30–33]. In the previous research, the diagnosis of DVT and PE in elderly patients with hip fractures was generally based on a combination strategy including clinical symptoms, duplex ultrasound (DUS), and computer tomography pulmonary angiography (CTPA) [12, 34]. A series of examinations confirm the diagnosis, but also bring a heavy financial burden to the patient. To simplify the diagnostic procedure, other scholars tried to use D-dimer adjustment formulas or other specific thresholds of well-established biomarkers to more accurately diagnose or predict the VTE after fracture. For example, Niikura et al. [30] established D-dimer cutoff levels for VTE screening in patients with fractures caused by high-energy injuries and showed moderate or high accuracy (area under curve 0.7–1.0) for predicting a VTE; Wu et al. [18] used the typical age-adjusted D-dimer cutoff value (age × 0.01 mg/L) in patients with knee or hip arthroplasty, and the results showed it had a better value in predicting DVT than a traditional threshold. In this study, we have got a new coefficient related to age (0.02), demonstrating a significantly improved specificity in diagnosis of a DVT, aiding in safely excluding those without a DVT in a larger proportion.

Compared to previous thresholds, the new age-adjusted D-dimer cutoff value has a higher specificity of 61%, significantly higher than 37% when using the typical age-adjusted D-dimer cutoff value. The number of patients with a negative D-dimer result increases from 594 (22%) when the traditional threshold of 0.5 mg/L is used to 1293 (59%) when the new age-adjusted formula is used. By definition, the reduction in sensitivity is due to the raising of the threshold for higher specificity, which causes some patients to fall below the new threshold and causes them to change from true positive to false negative [35].

Elderly patients with hip fractures can benefit from this new age-adjusted D-dimer value. Righini’s study showed that the cost-effectiveness of applying traditional thresholds in older people over 80 was poor due to excessive DUS [36]. The efficiency of typical age-adjusted D-dimer cutoff value is limited, in our study, the specificity when using the age-adjusted D-dimer cutoff value is significantly higher than that when using the other two thresholds. The increased specificity of the D-dimer test and DVT excluded proportion reduce the number of patients who need further DUS and unnecessary anticoagulant therapy with consequent clinical. The potential benefit would be fewer long waits and frequent mobility, thus decreasing physical impairments.

D-dimer test must be integrated with clinical pre-test probability (PTP) scores (such as the Wells score or the revised Geneva score) and DUS, when it was used to diagnosis DVT in published studies [24]. But when using the new age-adjusted threshold, we believe that there is no need to consider PTP, because PTP is routinely used to screen the patients with low and moderate pretest probability [25, 37], elderly hip fracture patients usually have a moderate, or high pretest probability due to trauma, mobility limitations or other comorbidities. Moreover, the applicant effect of PTP is not good in clinical practice, and the rate of PTP in which clinicians’ adherence to standardized diagnostic procedures is as poor as 50% to 60% in prior studies [38, 39]. Simplified clinical assessment algorithms using the new age-adjusted formula and DUS can save time and energy for medical staff. The new age-adjusted D-dimer cutoff value should be applied to specific D-dimer assays. The wide diversities of D-dimer assays used in the published studies showed the difficulty of selecting unified reference ranges and clinical thresholds, because of the multiple combinations of monoclonal antibodies and different assay reagent, and diverse D-dimer assays had the substantial differences in analytical performance [35, 40, 41]. The age-adjustment formula should be established based on different D-dimer assays correspondingly, instead of being widely used without a second thought.

The new age-adjusted formula is suitable for the hip fracture patients older than 65 years old. After the patient undergoes the first D-dimer test (Immunofluorescence quantitative analysis), doctors evaluate the risk of thrombosis using this formula, that is, doctors compare the D-dimer value and the age-adjusted value ( age × 0.02 mg/L). It should be noted that these conditions including high-energy accident, long-term injured-limb immobility or the significant delay to admission, concomitant suspicion of PE, ongoing anticoagulant treatment, multiple fractures, acute episode (acute infection, acute heart failure, etc.), and malignancy seriously affect the level of D-dimer; therefore, this formula is not applicable to patients with these conditions. Thence, this formula also has some shortcomings, and its general applicability is poor and it can only be applied to a limited group of people.

## Limitation

There are several limitations of our study. It is a single-center retrospective study with inherent defects. The retrospective data extraction may cause inaccurate information. The study is limited to elderly patients with hip fractures, so the results may not have excellent applicability in the general population. We don't evaluate patients who only undergo the D-dimer test or DUS. Likely, patients with highly reasonable suspicion of DVT go straight to perform DUS, resulting in a lower calculated incidence of DVT than that in nature. The D-dimer assays are heterogeneous in different researches. Our study only adopts one laboratory testing method, and how effective is the new age-adjusted cutoff value when other D-dimer assays are used, is unclear. A large multi-center prospective study should be conducted before the application of the new age-adjusted cutoff value which comes from our exploration.

## Conclusions

In this study, we developed a new age-adjusted D-dimer cutoff value (age × 0.02 mg/L) for a specified high-risk population of patients aged 65 years or older with hip fractures, and demonstrated the improved utility of the D-dimer test for exclusion of DVT. This formula can be considered for use in elderly hip fracture patients who meet the applicable standards as preoperative DVT screening, after its validity is confirmed by more well-evidenced studies.

## Data Availability

All the data used are available from the corresponding author on motivated requests.

## Abbreviations

DVT: Deep vein thrombosis
PE: Pulmonary embolism
VTE: Venous thromboembolism
ROC: Receiver operating characteristic curves
PPV: Positive predictive values
NPV: Negative predictive values
DUS: Duplex ultrasound
PTP: Pre-test probability
